# Study of the Differentially Expressed Genes in the *Pomacea canaliculata* Transcriptome after Treatment with Pedunsaponin A

**DOI:** 10.3390/metabo9110268

**Published:** 2019-11-06

**Authors:** Chunping Yang, Tianxing Lv, Yangyang Zhang, Bin Wang, Xiaomin Zhao, Min Zhang, Guoshu Gong, Xiaoli Chang, Guizhou Yue, Xiaoyan Qiu, Liya Luo, Huabao Chen

**Affiliations:** 1 College of Agronomy, Sichuan Agricultural University, Chengdu 611130, China; chunping79@163.com (C.Y.); mingyunzhefu@163.com (T.L.); zyy57sicau@163.com (Y.Z.); wbsicau@163.com (B.W.); zhaoxiaomin9426@163.com (X.Z.); yalanmin@126.com (M.Z.); guoshugong@126.com (G.G.); xl_changkit@126.com (X.C.); qiuxiaoyan0712@163.com (X.Q.); luoliya_sicau@163.com (L.L.); 2 College of Science, Sichuan Agricultural University, Chengdu 611130, China; yueguizhou@sicau.edu.cn

**Keywords:** pedunsaponin A, *Pomacea canaliculata*, transcriptome, DEGs

## Abstract

Transcriptomes, genomes, and proteomes have played important roles in the search for drug targets. To determine the molluscicidal mechanism of pedunsaponin A against *Pomacea canaliculata*, RNA-seq technology was adopted to analyze the differentially expressed genes (DEGs) in the *P. canaliculata* transcriptome after treatment with pedunsaponin A. As a result, 533 DEGs were identified, among which 255 genes were significantly upregulated and 278 genes were significantly downregulated. According to the analysis of Gene Ontology (GO) functions, we found that the DEGs were significantly enriched in the viral life cycle, UDP-glucose 4-epimerase activity, guanylate cyclase activity, the cyclic guanosine monophosphate (cGMP) biosynthetic process, and the cGMP metabolic process. The Kyoto Encyclopedia of Genes and Genomes (KEGG) pathway results showed that the DEGs were mainly involved in the hedgehog signaling pathway, phagosome, cytosolic DNA-sensing pathway, retinoic acid-inducible gene I like (RIG-I-like) receptor signaling pathway, bacterial secretion system, and nuclear factor-kappa B (NF-kappa B) signaling pathway. The above results indicated that pedunsaponin A causes a metabolic disorder, anomalous opening of membrane ion channels, and an imbalance in osmotic pressure between the interior and exterior of cells, eventually resulting in the death of cells involved in immune defense and influencing the immune response of *P. canaliculata*.

## 1. Introduction

*Pomacea canaliculata*, which belongs to the *Ampullarius* genus in the *Ampullariidae* family of *Mollusca*, is one of the most harmful alien invasive species in China. These snails have been threatening agricultural production, ecological security, and public health. *Pueraria peduncularis* belongs to the *Pueraria* genus in the *Leguminosae* family and is unique to China, where it is used in traditional Chinese medicine. The Biorational Pesticide Laboratory of Sichuan Agricultural University performed a series of experiments on the molluscicidal activity of *P. peduncularis*. The results showed that the extract of *P. peduncularis* had strong toxic effects on the snails, and that the main active component was pedunsaponin A, which exhibited an median lethal concentration (LC_50_) value of 3.893 mg·L^−1^ [1]. According to the observation of histopathological changes in the snails’ organs after treatment with pedunsaponin A by using histological sectioning and scanning electron microscopy techniques, we found that pedunsaponin A first causes the loss of cilia and then enters the hemolymph, destroys the structure of hemocytes, and influences the hemocyte count [2]. Furthermore, the cell membrane and cytoskeleton of *P. canaliculata* hemocytes were damaged, which led to an increase in cell mortality, dysfunction, cell cycle abnormalities, and apoptosis, and eventually resulting in the death of the snails [3]. However, its molecular mechanism was not clearly demonstrated.

A large number of sequence data are available from the transcripts in organisms to generated in studies on the regulation of gene expression by using RNA-seq technology; thus, an increasing number of scholars have applied this technology to identify drug targets through the analysis of differentially expressed genes (DEGs) after treatment with drugs. For example, Geng et al. [4] used *Gyradactylus kobayashii* parasitizing goldfish as the study object, and applied RNA-seq technology to study the pesticidal mechanism of mebendazole against *G. kobayashii*. The results showed that there were 141 DEGs of *G. kobayashii* after treatment with mebendazole. Combined with the real-time PCR data, it was speculated that four genes (*TF002*, *TF004*, *TF005*, and *TG004*) were closely related to the pesticidal mechanism of mebendazole against *G. kobayashii*. Hsu et al. [5] analyzed the toxic effects of triadimefon on zebrafish embryos and found that 3508 genes were significantly upregulated and that 88 genes were significantly downregulated after treatment with triadimefon. All the DEGs participated in signal transduction, and most of these genes were involved in cytochrome P450 enzymes and molecular metabolism. To determine the molluscicidal mechanism of pedunsaponin A against *P*. *canaliculata*, we analyzed the total RNA of *P. canaliculata* by using RNA-seq technology, compared the DEGs of the treated group with the control group, and analyzed the DEG functions in this study. As a result, this research provides references for determining the target of pedunsaponin A in *P. canaliculata* at the transcription level.

## 2. Results

### 2.1. Results of RNA-Seq and Sequence Shearing

A total of 441,206,324 original sequences were obtained by sequencing the transcriptomes of six samples of *P. canaliculata*. After quality evaluation for sequencing, there were 425,807,558 nonredundant sequences. As shown in Table 1, the error rate was 0.0147%, the average percentage of the bases with Phred values greater than 20 or 30 accounted for 97.05% and 92.29%, respectively, of the total bases, and the G and C bases accounted for 45.98%. The above results show that the quality and data volume of transcriptome sequencing were relatively high, thus meeting the requirements for subsequent data assembly and processing.

### 2.2. The Results of De Novo Assembly

We obtained 89,638 transcripts through de novo assembly of nonredundant sequences, and a total of 184,800,908 bases, which contained 42.38% G+C bases. The average length of transcripts was 2061.64 bp, and the N50 was 3385 bp. The longest transcript was as a unigene; 48,567 unigenes were obtained. Among these unigenes, the total number of bases was 85,118,502, the content of G+C bases was 42.23%, the average length was 1752.6 bp, and the N50 was 3135 bp (Table 2).

**Figure 1:**
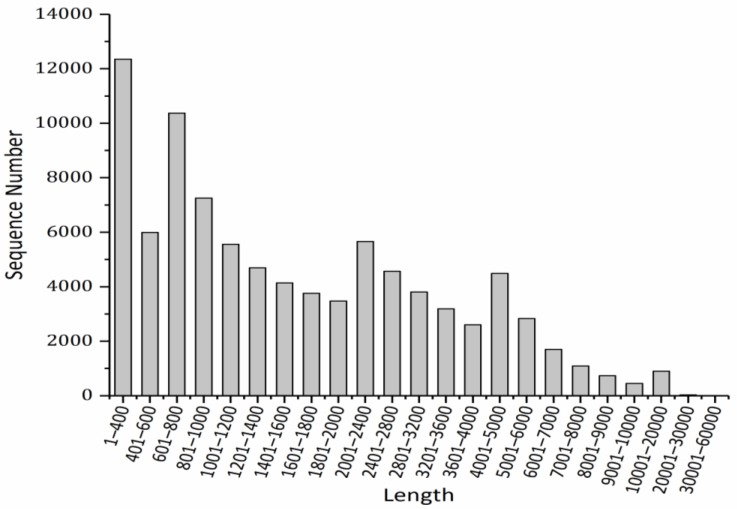
Length distribution of the assembled transcripts.

**Figure 2:**
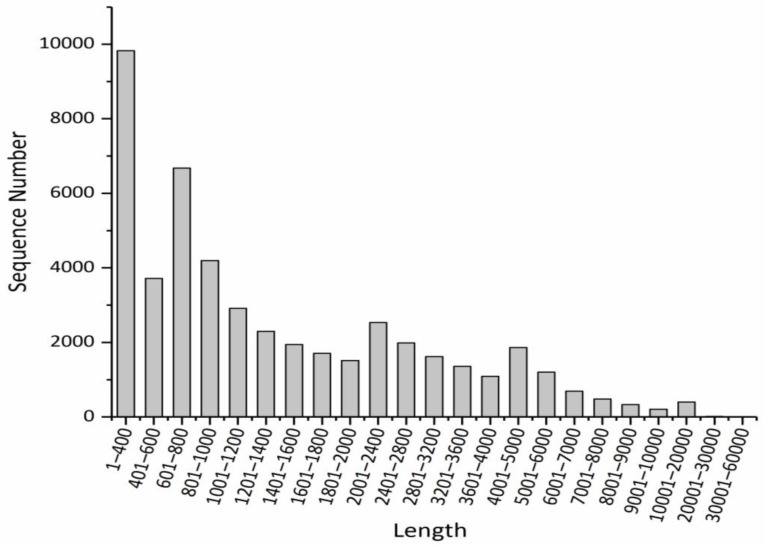
Length distribution of the assembled unigenes.

Length distribution analyses of the assembled transcripts and unigenes were carried out. As shown in Figure 1, transcripts with lengths ranging from 1 to 400 bp were the most common (13.78%), followed by those of 601 to 800 bp (11.57%). In Figure 2, similar results are shown but with different proportions, and the top two categories accounted for 20.23% and 13.75% of the transcripts, respectively. The above results indicated that the transcripts of *P. canaliculata* mainly consist of small or medium-sized fragments.

### 2.3. Functional Annotation of P. canaliculata Unigenes

According to the functional annotation results of *P. canaliculata* unigenes, there were 8751 unigenes that were matched to the Kyoto Encyclopedia of Genes and Genomes (KEGG) database (18.02%), and 6020 unigenes that were matched to the Gene Ontology (GO) database (12.40%) (Table 3).

#### 2.3.1. The Results of GO Annotation

**Figure 3:**
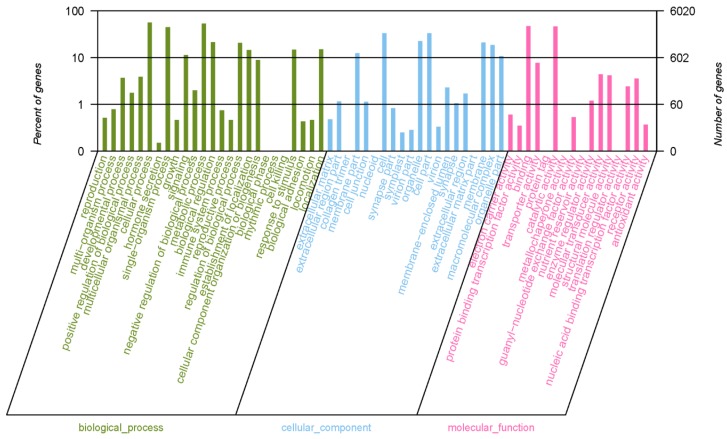
Second-level GO classification of *Pomacea canaliculata* unigenes.

The GO annotation results for *P. canaliculata* unigenes are shown in Figure 3. The identified GO functions were primarily focused on cellular processes, metabolic processes and single-organism processes in the biological process category, which accounted for 56.50%, 53.59%, and 45.03%, respectively, of the total unigenes. In the cellular component category, the unigenes were mainly related to the cell, cell part, organelle, and membrane terms, which accounted for 33.26%, 33.26%, 22.44%, and 20.83%, respectively, of the total unigenes. In the molecular function category, binding and catalytic activity accounted for the highest proportions of unigenes, at 47.09% and 46.40%, respectively.

#### 2.3.2. The Results of KEGG Annotation

**Figure 4:**
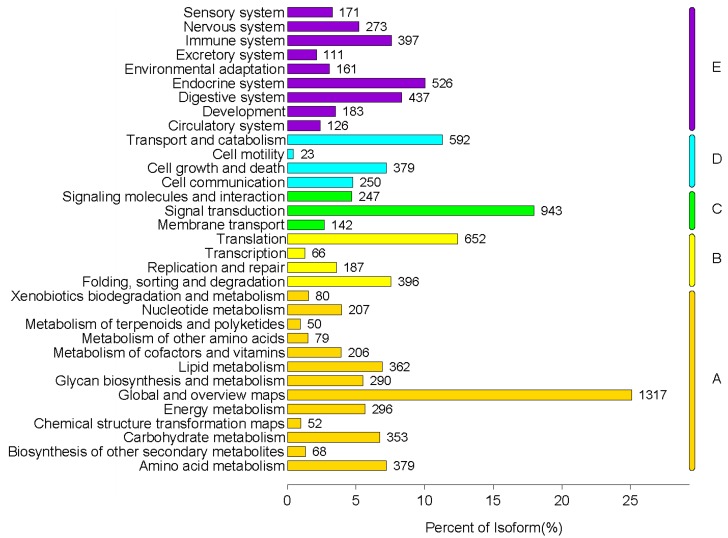
The KEGG pathway classification of *P. canaliculata* unigenes. (**A**) Metabolism; (**B**) Genetic Information Processing; (**C**) Environment Information Processing; (**D**) Cellular Process; (**E**) Organismal System.

The KEGG annotation results for the *P. canaliculata* unigenes showed that 8751 unigenes were mapped to 369 KEGG pathways. The unigenes could be classified into five branches according to the associated KEGG pathways, as shown in Figure 4.

### 2.4. The Analyses of DEGs

**Figure 5:**
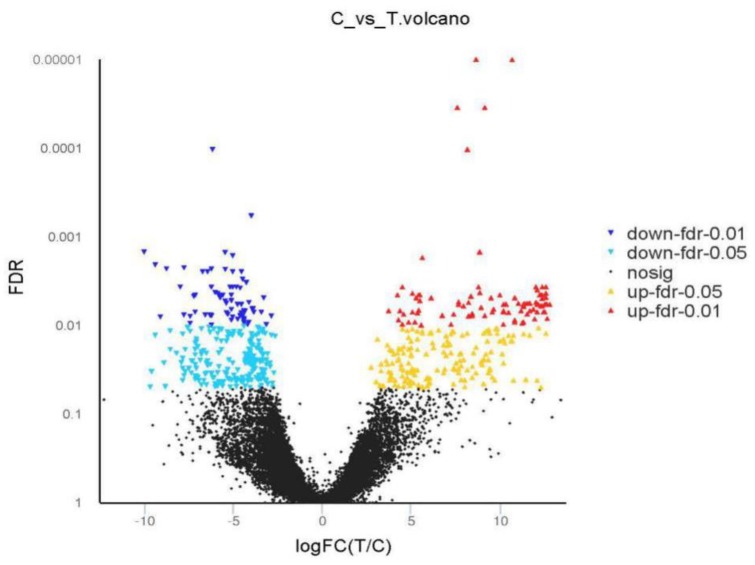
The visualization of differential expression gene. Red dot: Significantly upregulated gene; Blue dot: Significantly downregulated gene; Black dot: Not significantly differentially expressed gene. FDR: false discovery rate; FC: fold change.

Based on the calculation and screening results, 34970 DEGs were identified after treatment with pedunsaponin A compared with the blank control, among which 16831 genes were upregulated and 18139 genes were downregulated. Among these DEGs, 533 genes were significantly differentially expressed, with 255 genes being significantly upregulated and 278 genes being significantly downregulated (Figure 5).

#### 2.4.1. GO Enrichment Analyses of DEGs

**Figure 6:**
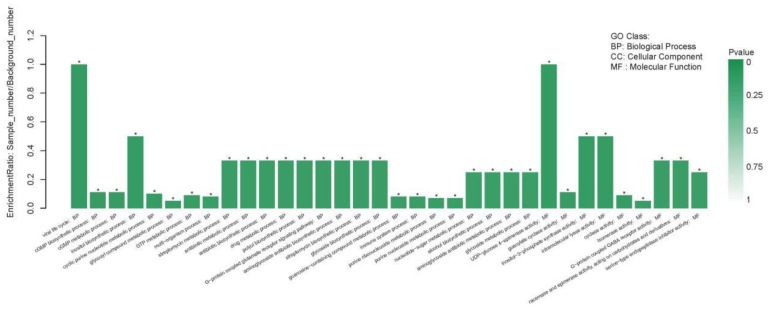
The GO enrichment situation of differentially expressed genes (DEGs).

According to the analysis results, the DEGs were significantly enriched in both biological processes and molecular functions (Figure 6). In the biological process category, the DEGs were significantly enriched in 25 GO terms: viral life cycle, cyclic guanosine monophosphate (cGMP) biosynthetic process, cGMP metabolic process, inositol biosynthetic process, cyclic purine nucleotide metabolic process, glycosyl compound metabolic process, GTP metabolic process, multi-organism process, streptomycin metabolic process, antibiotic metabolic process, antibiotic biosynthetic process, drug metabolic process, polyol biosynthetic process, G-protein coupled glutamate receptor signaling pathway, aminoglycoside antibiotic biosynthetic process, streptomycin biosynthetic process, glycoside biosynthetic process, guanosine-containing compound metabolic process, immune system process, purine ribonucleoside metabolic process, purine nucleoside metabolic process, nucleotide-sugar metabolic process, alcohol biosynthetic process, aminoglycoside antibiotic metabolic process, and glycoside metabolic process.

In the molecular function category, the DEGs were significantly enriched in 10 GO terms: UDP-glucose 4-epimerase activity, guanylate cyclase activity, inositol-3-phosphate synthase activity, intramolecular lyase activity, cyclase activity, isomerase activity, G-protein coupled GABA receptor activity (racemase and epimerase activity, acting on carbohydrates and derivatives), and serine-type endopeptidase inhibitor activity.

#### 2.4.2. The KEGG Pathway Annotation Results of the DEGs

**Figure 7:**
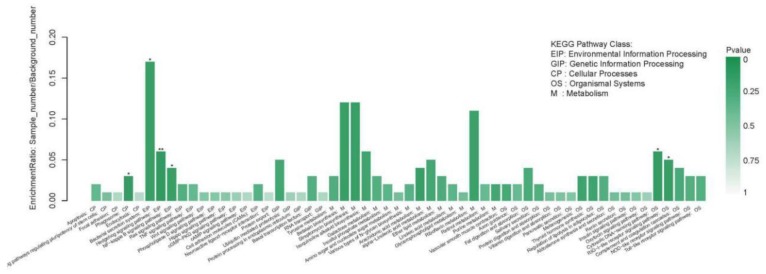
The KEGG pathway enrichment situation of DEGs.

The KEGG pathways of DEGs were annotated, and the results showed that the DEGs were mainly annotated to 58 pathways, among which six pathways were significantly enriched, including the hedgehog signaling pathway, phagosome, cytosolic DNA-sensing pathway, retinoic acid-inducible gene I like (RIG-I-like) receptor signaling pathway, bacterial secretion system, and the NF-kappa B signaling pathway (Figure 7).

## 3. Discussions

In recent years, with the development of molecular biotechnology there has been research related to the search for drug targets based on transcriptome, genome, and proteome technologies, promoting the process of discovering drug targets at the biomacromolecular level. At present, research on the genome and transcriptome of *P. canaliculata* is still in a nascent stage, and RNA-seq technology is a powerful tool for studying these snails, whose genome has not yet been mapped and for which data are scarce [6]. To determine the molluscicidal mechanism of pedunsaponin A against *P. canaliculata* at the molecular level, we performed the whole-transcriptome sequencing of *P. canaliculata* in this study. Compared with the blank control snails, 533 DEGs were identified after treatment with pedunsaponin A, among which 255 genes were significantly upregulated and 278 genes were significantly downregulated. The results showed that pedunsaponin A could influence the gene expression of *P. canaliculata*.

Based on the GO enrichment analyses of the DEGs, we found that the DEGs were significantly enriched in the viral life cycle, UDP-glucose 4-epimerase activity, guanylate cyclase activity, cGMP biosynthetic process, and cGMP metabolic process categories. The enzyme UDP-glucose 4-epimerase catalyzes the freely reversible interconversion of UDP-glucose to UDP-galactose. As critical precursors for carbohydrate metabolism, UDP-glucose and UDP-galactose play important roles during biosynthetic and metabolic pathways [7,8]. There are two main types of guanylate cyclase (GC): membrane GC (mGC) and soluble GC (sGC). mGC, which is a transmembrane protein, can carry out the membrane transport of the molecules through its specific folding and bending types when reacting to a particular molecule. sGC can catalyze the synthesis of the second messenger cGMP from guanosine triphosphate (GTP), and cGMP can then activate downstream effector molecules such as phosphodiesterase, cyclic nucleotide-gated ion channels, and protein kinase G, and plays a key role in the gastrointestinal system, blood circulation, and nervous system [9,10]. These results demonstrate that pedunsaponin A can disrupt material and energy exchange during *P. canaliculata* growth and affect the cell osmotic pressure balance by regulating gene expression related to metabolism and ion channels in the snail.

Different gene products act in coordination with each other to perform biological functions in organisms; therefore, the KEGG pathway analysis of DEGs is conducive to further interpreting the functions of genes [11]. According to the results of KEGG annotation, we found that the DEGs were significantly enriched in the hedgehog signaling pathway, phagosome, cytosolic DNA-sensing pathway, RIG-I-like receptor signaling pathway, bacterial secretion system, and NF-kappa B signaling pathway. These pathways play an important role in maintaining internal and external balance, the host immune response, and the cell proliferation and apoptosis of *P. canaliculata* [12,13,14,15,16,17,18]. The results of the GO enrichment analyses of DEGs were similar to KEGG pathway annotations.

Based on the above results, we speculated that certain genes of *P. canaliculata* are differentially expressed after treatment with pedunsaponin A, resulting in a metabolic disorder in the snail, effects on crucial regulatory factors of growth and development, and the abnormal opening of iron channels in the cell membrane, which could interrupt the osmotic balance in cells, cause the death of hemocytes involved in the immune defense, and further affect the host immune response. Modern genetic studies have indicated that most genes function through their protein products and that cells rely on multiple metabolic and regulatory pathways to survive. Therefore, we cannot obtain a full understanding of the toxicity mechanism of pedunsaponin A against *P. canaliculata* by analyzing only the DEGs, and further proteomic studies are needed to search for their target proteins.

## 4. Materials and Methods

### 4.1. Materials

*P. canaliculata* was collected from the paddy field of Sichuan Agricultural University in Chengdu City, Sichuan Province, China (30.71°N, 103.87°E). The collected snails were cleaned and kept in plastic boxes (40 cm length × 25 cm width × 30 cm height) containing a certain volume of distilled water. The boxes were covered with nylon nets and placed in the laboratory. The snails were given clean water and fed with cabbage, and dead snails were removed every day. The snails were acclimatized to the laboratory conditions for 5 days before the experiments, and snails weighing 5 ± 0.5 g were selected for the experiments.

Pedunsaponin A (purity above 98%) was extracted in the Biorational Pesticide Laboratory of Sichuan Agricultural University. First, the roots of *P. peduncularis* were collected from wild populations in Yaan City, Sichuan Province, China (29.90°N, 102.92°E), and air-dried. Then, root compounds were extracted from the ground roots with 90% ethanol, and the organic solvent was concentrated to obtain the crude extract of *P. peduncularis*. Finally, the crude extract was isolated, purified, and analyzed using a C18 column and high-performance liquid chromatography (HPLC). As a result, pedunsaponin A with a purity greater than 98% was obtained.

### 4.2. Methods

#### 4.2.1. Sample Collection and RNA Extraction

Ten snails were selected and treated with 20 µg·mL^−1^ pedunsaponin A for 48 h. Distilled water was used as a blank control, and three replicates were performed for the experiment. After processing, *P. canaliculata* tissues were collected on ice during the experiments, after which the tissue samples were quickly frozen with liquid nitrogen and stored at −80 °C. The total RNA of the tissue samples was extracted with the Trizol method, and a Nanodrop2000 spectrophotometer was used to detect the concentration and purity of the RNA, agarose gel electrophoresis was used to check the integrity of the RNA, and an Agilent 2100 Bioanalyzer was used to determine the RIN (RNA Integrity Number) value.

#### 4.2.2. RNA-Seq

**Figure 8:**
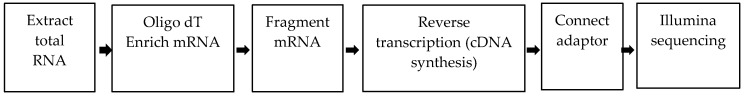
The procedure of Illumina sequencing.

Qualified RNA samples were treated as shown in Figure 8, and then sequenced by using an IlluminaHiseq sequencer. As a result, the sequencing data of *P. canaliculata* (treated with distilled water or pedunsaponin A, respectively) were obtained.

#### 4.2.3. Sequence Shearing and de Novo Assembly

The original image data, which were sequenced by Illumina, were converted to sequential data (FASTQ format) by base calling with the Phred program, and the original sequencing data files were obtained [19]. Then, the original sequencing data were sheared by using the software SeqPrep (https://github.com/jstjohn/SeqPrep) and Sickle (https://github.com/najoshi/sickle) to obtain the clean data. Due to the lack of a reference genome for *P. canaliculata*, the de novo assembly of high-quality sequences was conducted by using Trinity software (http://trinityrnaseq.sourceforge.net/), and high-quality transcripts were obtained. Subsequently, the longest transcript was taken as a unigene [20], and the indexes (total number, base number, GC%, average length, N50 value) of the transcripts/unigenes were analyzed with statistical methods.

#### 4.2.4. Annotation of Unigene Functions

Gene prediction for the unigene sequences was carried out with Trinity software, and the prediction results were subsequently corrected via the Pfam (http://pfam sanger.ac.uk/) database. All nucleotide sequences were compared against the NR and KEGG databases using BlastX. Finally, the obtained annotation results were converted into Gene Ontology (GO) annotations by Blast2GO (http://www.blast2go.com/b2ghome).

#### 4.2.5. Screening and Analysis of DEGs

RSEM software (http://www.biomedsearch.com/nih/RSEM-accurate-transcript-quantification-from/21816040.html) was applied to analyze gene expression from the *P. canaliculata* transcripts, and snails that were treated with distilled water were used as a control to screen the significant DEGs of the snails that were treated with pedunsaponin A with the software edgeR (http://www.bioconductor.org/packages/2.12/bioc/html/edgeR.html) [21]. Furthermore, GO (http://www.geneontology.org) function and KEGG (http://www.genome.jp/kegg/) pathway annotations were conducted with the software Goatools (https://github.com/tanghaibao/GOatools) and KOBAS (http://kobas.cbi.pku.edu.cn/home.do) [22,23].

## Figures and Tables

**Table 1:** Illumina high-throughput sequencing (IlluminaHiseq) data and the quality evaluation of the *Pomacea canaliculata* sequences.

Sample Name	Original Sequence Number	Nonredundant Sequence Number	Error%	Q20%	Q30%	GC%
C_1	83351566	80289528	0.0149	96.96	92.1	45.47
C_2	71588586	69052548	0.0148	96.99	92.14	46.94
C_3	65875322	63312812	0.015	96.94	91.99	47.55
T_1	74871166	72325846	0.0146	97.07	92.35	45.26
T_2	68095074	65957654	0.0144	97.17	92.61	45.13
T_3	77424610	74869170	0.0144	97.14	92.54	45.55
Average	73534387	70967926	0.0147	97.05	92.29	45.98
Total	441206324	425807558	—	—	—	—

Note: C indicates control group; T indicates treatment group; Q20% and Q30% indicate the percentages of bases in which the Phred values are greater than 20 and 30, respectively; GC% indicates the percentage of the sum of G and C bases among total bases.

**Table 2:** Statistics of the sequencing assembly of the transcriptome of *P. canaliculata*.

Type	Total Number	Base Number	GC (%)	Average Length (bp)	N50 (bp)
transcripts	89,638	184,800,908	42.38	2061.64	3385
unigenes	48,567	85,118,502	42.23	1752.6	3135

Note: N50 indicates the length of corresponding transcript, which cumulative transcripts length reaches half of the total length of transcripts (assembled transcripts are arranged from largest to smallest by the length) after adding it.

**Table 3:** Statistics of the unigene annotation information.

Database	Total Number of Unigenes	Annotated Unigenes	Percentage (%)
Kyoto Encyclopedia of Genes and Genomes (KEGG)	48,567	8751	18.02
Gene Ontology (GO)	48,567	6020	12.40
